# Post-Translational Modifications of Retroviral HIV-1 Gag Precursors: An Overview of Their Biological Role

**DOI:** 10.3390/ijms22062871

**Published:** 2021-03-11

**Authors:** Charlotte Bussienne, Roland Marquet, Jean-Christophe Paillart, Serena Bernacchi

**Affiliations:** Architecture et Réactivité de l’ARN, UPR 9002, IBMC, CNRS, Université de Strasbourg, 2 allée Konrad Roentgen, CEDEX F-67084 Strasbourg, France; c.bussienne@ibmc-cnrs.unistra.fr (C.B.); r.marquet@ibmc-cnrs.unistra.fr (R.M.); jc.paillart@ibmc-cnrs.unistra.fr (J.-C.P.)

**Keywords:** HIV-1, Pr55^Gag^ precursor, post-translational modifications, retroviral Gag precursors, retroviral life cycle

## Abstract

Protein post-translational modifications (PTMs) play key roles in eukaryotes since they finely regulate numerous mechanisms used to diversify the protein functions and to modulate their signaling networks. Besides, these chemical modifications also take part in the viral hijacking of the host, and also contribute to the cellular response to viral infections. All domains of the human immunodeficiency virus type 1 (HIV-1) Gag precursor of 55-kDa (Pr55^Gag^), which is the central actor for viral RNA specific recruitment and genome packaging, are post-translationally modified. In this review, we summarize the current knowledge about HIV-1 Pr55^Gag^ PTMs such as myristoylation, phosphorylation, ubiquitination, sumoylation, methylation, and ISGylation in order to figure out how these modifications affect the precursor functions and viral replication. Indeed, in HIV-1, PTMs regulate the precursor trafficking between cell compartments and its anchoring at the plasma membrane, where viral assembly occurs. Interestingly, PTMs also allow Pr55^Gag^ to hijack the cell machinery to achieve viral budding as they drive recognition between viral proteins or cellular components such as the ESCRT machinery. Finally, we will describe and compare PTMs of several other retroviral Gag proteins to give a global overview of their role in the retroviral life cycle.

## 1. Introduction

Post translational modifications (PTMs) introduce a vast diversity in proteome including addition of chemical groups, like phosphorylation, methylation, acetylation, redox-based modifications, or alternatively, addition of polypeptides like ubiquitination, sumoylation or ISGylation. PTMs thus play a key role in functional proteomic by regulating proteins activity, their localization, and the interaction with cellular or viral factors. Even though many proteins are modified shortly after translation, PTMs can also occur at different steps such as after protein folding or protein re-localization to influence their biological activity at those specific sites (for reviews see [1,2]). Besides, depending on the nature of the modification, they can also finely tune reversible processes. Consequently, analysis of PTMs can provide an invaluable insight into cellular functions.

Viruses rely on the protein synthesis machinery of the host to support the production of viral progeny, and several cellular pathways are modulated by viruses to achieve the critical steps in viral replication. Hence, it is not surprising that viruses developed different strategies to either counteract or exploit PTMs of cellular factors, and that many viral proteins carry PTMs. Interestingly, PTMs are strongly involved in the regulation of different steps of the retrovirus viral cycle (for reviews see [3,4]). More specifically, in the HIV-1 (human immunodeficiency virus type 1) context, PTMs within the 55-kDa viral precursor, Pr55^Gag^ (or Gag), were found to be necessary for regulating the last phase of the viral cycle, leading to the assembly of viral particles. Besides, several pieces of evidence have shown that other retroviral Gag carry various PTMs regulate viral replication and pathogenesis. This review will summarize our current knowledge on PTMs observed in HIV-1 Pr55^Gag^ and in other retroviral Gag proteins. Considering the role of the PMTs in the retroviral life cycle, the analysis of PTMs in retroviral Gag precursors could be particularly important for a deeper understanding of the molecular mechanisms driving retroviral replication. In a further step, this knowledge could contribute to the identification of new targets, and the design of new treatments against retroviral replication.

## 2. HIV-1 Pr55^Gag^

**Figure 1:**
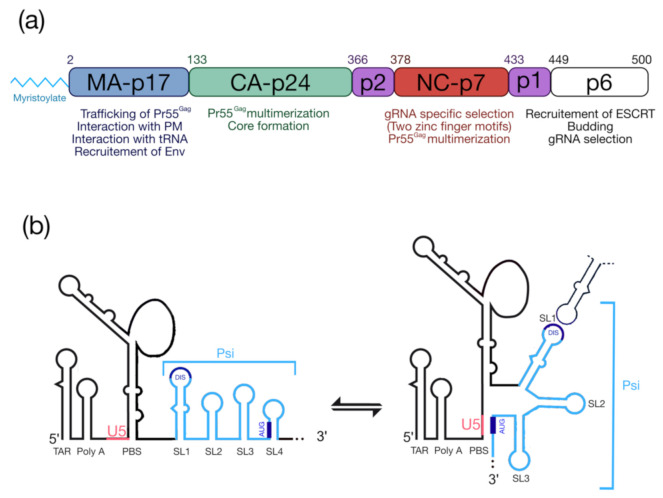
Pr55^Gag^ and the 5′UTR of HIV-1 genomic RNA. (**a**) Functional domains of Pr55^Gag^ and a short description of their roles. (**b**) Schematic representation of the secondary structure model of the 5′UTR (adapted from [29]). TAR: transactivation response element; Poly-A: 5′-copy of the polyadenylation signal; PBS: Primer Biding Site; DIS: Dimerization Initiation Site; Psi: packaging signal spanning SL1 to SL4; U5: unique region at the 5′ end. The structure represents the U5-AUG conformation [5,6].

The HIV-1 Pr55^Gag^ precursor (Figure 1a) plays a crucial for genomic RNA (gRNA) packaging, since it specifically selects the full-length gRNA amongst many other RNAs (cellular and spliced viral RNAs) and this process involves specific interactions between Pr55^Gag^ and the highly structured 5′ region of the gRNA [5,6], which contains the packaging signal (Psi) spanning SL (stem-loop)1 to SL4 in the 5′-end region of gRNA [7,8,9] (Figure 1b). In cells, the HIV-1 gRNA dimer in association with low-order Pr55^Gag^ multimers [10,11,12] forms a viral ribonucleoprotein complex that traffics to the plasma membrane (PM) where the assembly of the viral particle occurs (for reviews see [13,14,15]). HIV-1 Pr55^Gag^ is composed of four structural domains named matrix (MA), capsid (CA), nucleocapsid (NC), p6, and two spacer peptides (p2 and p1) (Figure 1a) [16] and each of them carry PTMs.

From the N-terminus, the 17 kDa MA domain that possesses a bipartite signal leads to Pr55^Gag^ interaction with the PM. The first signal corresponds to the N-terminal myristoylated Glycine 2 (G2) (see § “HIV-1 Pr55^Gag^ Myristoylation”), while the second one is constituted by a highly basic region (HBR) at the MA surface (for a review see [17]). MA was also found to interact with nucleic acids such as host tRNAs [18], and recent findings showed that MA-RNA binding ensures the specific interaction between Pr55^Gag^ and the PM, by preventing nonspecific binding of Gag to intracellular membranes [19,20]. The CA is a 24 kDa domain that drives Pr55^Gag^ multimerization and leads to formation of the viral core [21,22,23]. Next, NC is a 7 kDa domain, which is crucial for specific interaction with gRNA and for the incorporation of tRNA^Lys3^, which is the primer for reverse transcription. NC displays two zinc finger motifs (CCHC) that specifically interact with the Psi (Figure 1b) [24,25]. This domain also contributes to Pr55^Gag^ multimerization thus promoting viral assembly [26,27,28]. At the C-terminal end of Pr55^Gag^, the unstructured p6 domain of 6 kDa is required for specific binding to the gRNA [29], and is involved in the recruitment of the ESCRT (Endosomal Sorting Complex Required for Transport) machinery that regulate viral particle budding. Finally, Pr55^Gag^ codes for two spacer peptides, sp1 and sp2 (also named p2 and p1, respectively), regulating the kinetics of Pr55^Gag^ maturation.

The next sections of this revue will describe which PMTs are carried by the different Pr55^Gag^ domains and what are their roles in the viral life cycle.

## 3. HIV-1 Pr55^Gag^ Myristoylation

**Figure 2:**
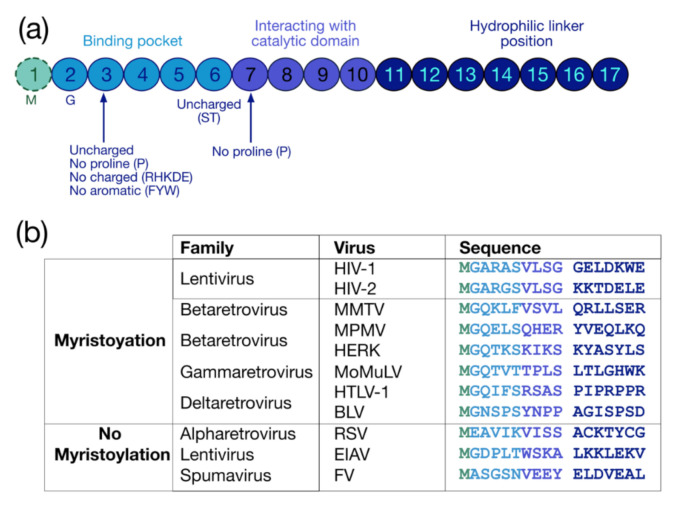
Protein sequence required for myristoylation and sequences of retroviral myristoylated MA domains. (**a**) Pro-myristoylated consensus sequence underlying the three regions regulating myristoylation: the binding pocket (positions 1–6), the catalytic domain (positions 7–10) and the hydrophilic linker (positions 11–17) [34,35]. (**b**) Comparison of the first 17 residues of myristoylated MA domains in different retroviruses. Myristoylation is generally conserved in retroviruses such as lentivirus (HIV-1), betaretrovirus (Mason-Pfizer monkey virus (MPMV), mouse mammary tumor virus (MMTV), and human endogenous retrovirus type K (HERK)), gammaretrovirus (Moloney murine leukemia virus (MoMuLV) and murine leukemia virus (MLV)), and deltaretrovirus (human T-lymphotropic viruses (HTLV-1) and bovine leukemia virus (BLV)), but not in alpharetrovirus (Rous sarcoma virus (RSV)), some other lentivirus (equine infectious anemia virus (EIAV)), and in spumavirus (foamy virus (FV)).

The myristoylation is a co-translational and irreversible modification consisting in the addition of a 14-carbon saturated fatty acid myristate to the protein via an amid bond by the N-myristoyl-transferase (NMT) (for reviews see [30,31,32]). The myristoylation can be achieved on an internal glycine (G) inside a consensus sequence recognized by NMTs, which is G-X2-X3-X4-(S/T/C)-X6 (Figure 2a). The G residue at the first position is necessary for this PMT, while at the second position there is preferentially an uncharged residue (except for proline (P)) or an aromatic amino acid. At the fifth position, uncharged residues are found, preferentially serine (S) and threonine (T) (for a review see [33]), while P is not accepted at the sixth position [34]. In sum, three regions finely regulate myristoylation: the binding pocket (positions from 1 to 6), the catalytic domain (positions from 7 to 10) and the hydrophilic linker (position from 11 to 17) [34,35] (Figure 2a).

Myristoylation is rather conserved in retroviruses (Figure 2b) (For reviews see [17,36] and [37,38]), and this PTM globally regulates the interaction of retroviral precursors with membranes and sub-membrane domains, such as lipid rafts. However, this modification is not sufficient by itself for membrane binding, and a distant polybasic domain is thus required to complete the optimal attachment of myristoylated proteins to the PM (for reviews see [39,40]). In HIV-1, this task is reached by the HBR spanning residues 17 to 31 of the MA domain, which contributes to a strengthening of the interaction with the PM thanks to electrostatic interactions with the negatively charged PI(4,5)P2 [41,42,43].

**Figure 3:**
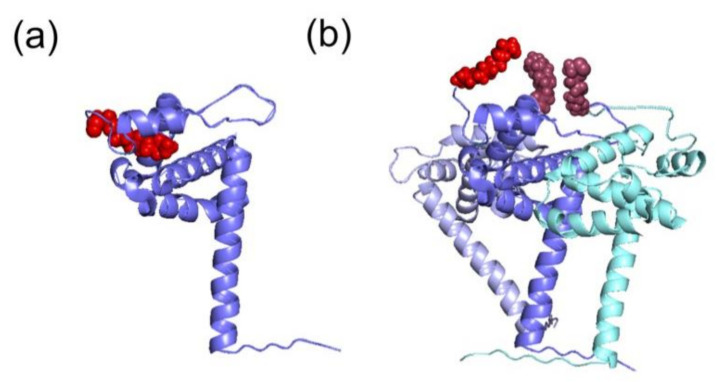
Different structural conformations of HIV-1 MA monomer or trimer. The tertiary structures of the MA domain in the different conformations of the switch look similar. (**a**) The MA domain in its monomeric conformation (in blue) displays a sequestered myristoyl group (in red) (PDB: 1UPH [36]). (**b**) Representation of the trimer of MA (in blue, light blue and cyan) and the corresponding exposed myristoyl groups (in red). This model was proposed according to which the myristoyl group is exposed in the multimeric form, thus allowing its interaction with PM (adapted from [37]).

The myristoyl moiety can be exposed or sequestered in the hydrophobic pocket of the mature MA (Figure 3a,b) by the so-called myristoyl-conformational switch [37,39,42,44,45], which controls the exposure of myristoyl group for insertion into the PM, thus contributing to the prevention of aberrant interactions with intracellular membranes. The myristate exposure was found to be triggered by the interactions occurring between Pr55^Gag^ and PI(4,5)P2 [41]. Besides, NMR studies demonstrated that myristate exposure is also regulated by the trimerization of the protein [37,42], and this would explain why the mature MA displays a lower affinity for membranes in comparison with the full-length precursor [37,41,42]. Indeed, several Pr55^Gag^ domains, such as CA, p2 and NC, contribute to the self-association of the precursor and, as a consequence, to the myristate exposure (Figure 3b) [37]. Accordingly, mutational experiments on these domains inhibiting Pr55^Gag^ multimerization, impair Pr55^Gag^ binding to the membrane [37].

## 4. Gag Myristoylation in Other Retroviruses

The MA domains of retroviral Gag polyproteins display two main roles: they participate in genome incorporation, as several analyses recently pointed out, and they are implicated in membrane association. Interestingly, the majority of the retrovirus family displays a myristoylated MA domain (for reviews see [46,47,48,49]).

Among the different genera in which MA is myristoylated, the genus gammaretrovirus is composed by simple and oncogenic retroviruses. One representative virus of this family is MLV inducing leukemias or lymphomas in mice [50]. The MA domain of the primary form of MLV Gag, Pr65^Gag^, is myristoylated and contains a polybasic region in its globular domain that interacts electrostatically with PI(4,5)P2 at the PM, similarly to HIV-1 [47]. Besides, MLV has the particularity of encoding an additional form of Gag, gPr80^Gag^, which is glycosylated, but not myristoylated, and this last one is involved in the Pr65^Gag^ trafficking to the PM [51]. However, beside MLV budding at PM, intracellular budding events can also occur into multivesicular bodies (MVBs) or in intracellular compartments as late endosomes in which virus-like particles (VLPs) accumulated [52]. Then, the deltaretrovirus genus contains complex and oncogenic retroviruses, and consists of two different groups, the primate T-lymphotropic viruses (PTLVs) including HTLV-1 and non-primate species, such as BLV [53,54,55]. Similarly, to MLV, the assembly of those retroviruses can occur at the PM, as well as in intracellular compartments such as late endosomes, MVBs or similar compartments [56]. The myristoylation of MA and the presence of basic amino acids leads to membrane binding and is, in this case, a PI(4,5)P2-independent process [57,58,59]. Indeed, the HTLV-I viral precursor Pr53^Gag^ is able to bind membranes by electrostatic interactions involving the zwitterionic phosphatidylcholines (PC) and the negatively charged phosphatidylserines (PS) contained in endocytic membranes [57,60]. Moreover, a model was proposed in which the HBR in the HIV-1 MA domain would bind RNA to prevent premature or non-specific binding to cellular membranes [19,20,61]. Interestingly, a similar regulation between MA and RNA was proposed for BLV [62]. Conversely, the lack of this RNA regulation in HTLV-1 could explain the binding of myristoylated MA to the cellular membranes of intracellular compartments [57]. Finally, betaretroviruses show many similarities with lentiviruses, including a myristoylated MA domain [63]. This genus is composed of two groups: the first one is represented by MMTV [64] and by MPMV; and the second one is represented by HERK [65]. Myristoylated-deficient HERK Gag was observed to localize in the nucleus [66]. Contrary to other lentiviruses, NMR structures of MA domains of MPMV [63] and MMTV [67] show that the myristate group is hidden inside the MA in its oligomeric form. These differences suggest that betaretroviruses have developed different strategies to sequester the myristoyl group until the VLP is bound to the PM. At this site, a conformational change, leading to exposure of the myristate group would occur, similarly to other retroviral Mas that bind PM [67].

In contrast, some retroviral Gag precursors are not myristoylated. Indeed, alpharetroviruses represents simple and oncogenic retroviruses like RSV [68]. At the PM, RSV Pr76^Gag^ interacts with charged lipids PI(4,5)P2 [38,69], and to ensure proper Pr76^Gag^-PM association, the lack of myristoylation is then counterbalanced by electrostatic interactions occurring between the inositol phosphates and a membrane binding domain (MBD), which is composed of basic residues forming a patch of clustered lysines (K) and arginines (R) on the MA surface ([49,68,70], for a review see [71]). Similarly, the MA domain of the lentivirus EIAV [72,73] is not myristoylated, but binds preferentially with phosphatidylinositol 3-phosphate (PI(3)P) with a higher affinity compared to PI(4,5)P2 [73,74]. Finally, foamy viruses (FV) as the PFV presents interesting differences compared to HIV-1 ([75], for a review see [76]). In particular, the FV Pr74^Gag^ displays a limited number of PTMs compared to the other retroviruses, and strikingly, the FV MA domain contains neither the HBR nor a myristoylation modification. All those elements emphasize a different evolutionary history among retroviruses [75]. Indeed, in this case, viral Env proteins play a major role for viral budding, and the co-expression of Pr74^Gag^ with Env is necessary for VLP production [77].

In sum, there are three main distinct strategies used by retroviruses to target membranes for budding. The first one requires the myristate exposure and a highly basic region (HBR) in the MA domain of retroviral precursors to interact with PM. The two others display dispensable myristoylation to achieve proper membrane binding since the hydrophobic interactions are in this case substituted by electrostatic ones produced by a basic domain in the MA, or alternatively by interactions between the precursor and viral elements such as Env proteins.

## 5. HIV-1 Pr55^Gag^ Phosphorylation

**Figure 4:**
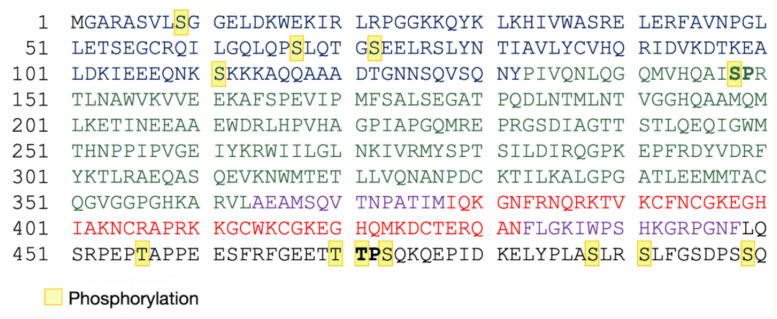
Phosphorylated residues in HIV-1 Pr55^Gag^. The different colors represent the Pr55^Gag^ domains, MA (blue), CA (green), spacer peptides p1 and p2 (purple), NC (red), and p6 (black). Phosphorylation positions are highlighted in yellow. TP (in p6) and SP (in CA) motifs involved in the ERK2 recruitment and incorporation into viral particle are indicated in bold [91,93,94,95,96].

Phosphorylation consists of the addition of a phosphate group to the side chain of amino acids. This PMT modifies the local electrostatic potential of proteins, induces conformational modifications, and affects the protein subcellular localization (for a review see [78], and [79,80]). Kinases, which are the enzymes that catalyze the transfer of phosphate group, have a role at multiple steps of HIV-1 viral, and the inhibition of cellular kinases interacting with HIV-1 at the nuclear level has been shown to affect the viral replication cycle [81]. Among HIV-1 viral proteins, which are phosphorylated, there is Pr55^Gag^ (Table 1 and Figure 4). The MA domain is a substrate for the protein kinase C (PKC) [82], which catalyzes S and T phosphorylation. Several studies identified S111 in HIV-1 MA as the substrate for PKC [82]. Interestingly, substituting S111 with an alanine (A) led to decreased association of Pr55^Gag^ with PM, even though MA was myristoylated. This suggests that PKC could also be involved in membrane binding by regulating the exposure of the myristoyl group [83,84].

Alpha-screen assays allowed us to screen for human kinases interaction with the HIV-1 precursor, and the p6 domain resulted to be a target for PKC. In a further step, mass spectrometry indicated the phosphorylation of S488 residue [85,86]. Its substitution with a hydrophobic aromatic residue such as phenylalanine (F), which can occur spontaneously during anti-retroviral treatments, was found to perturb CA-SP1 processing, virus morphogenesis, maturation and virion infectivity [87,88,89]. On the other hand, the substitution of S488 by another non phosphorylable residue, such as asparagine (N), displayed no global impact on infectivity, thus suggesting that the production of non-mature viral particles would not be due to the lack of phosphorylation, but by the substitution itself [89]. Moreover, the phosphorylation of the p6 domain was found to be also important for the recruitment of the viral factor Vpr. As a consequence, the inhibition of PKC activity reduced Vpr level in virions, and this affected HIV-1 infectivity [85]. The p6 domain is the main phosphorylated domain in Pr55^Gag^ and can be phosphorylated at several positions [86,90]. Indeed, phosphoamino acid analysis [90] and mass spectrometry experiments [86] identified several phosphorylated amino acids (Table 1 and Figure 4), that were found to globally promote viral budding [91]. Moreover, electron microscopy analysis revealed that mutation T471A leads to immature viral particles incompletely separated from PM, and immunoblotting analysis showed an incomplete Pr55^Gag^ proteolytic maturation [91]. In contrast, other findings showed no effects on assembly or on viral release when T471 was substituted with isoleucine (I) or N. Since none of these amino acids can be phosphorylated, it is possible that the observed differences were not due to phosphorylation itself. Furthermore, except for T456 located in the PTAP late domain, the other eleven positions that can be phosphorylated in the p6 domain present redundancy. Mutagenesis experiments confirmed that the modifications of those residues seem to be dispensable for viral release and infectivity [86].

Experiments using an inhibitor of cyclin-dependent kinases [92] showed that also a MAP kinase, the extracellular-signal-regulated kinase 2 (ERK2), is involved in p6 phosphorylation, and this factor can be incorporated into viral particles by interacting with the S148-P149 motif in CA and T471-P472 in p6 [91,93,94,95,96] (Table 1, Figure 4). HIV-1 particles without active ERK2 were found to be poorly infectious due to a defect in reverse transcription [93,95]. Interestingly, ERK2 phosphorylates other viral proteins including Rev [97], Nef [98], Vif [99,100], and mature MA [95]. Besides, the substitution of four highly conserved and major phospho-acceptor S residues in the mature MA (Table 1) with A was found to impair viral replication [95,101].

Finally, the tyrosine kinase Src can also be incorporated into HIV-1 particle [102], and it is involved in the phosphorylation of the tyrosine (Y) 132 in a minority of mature MA proteins. This PMT was shown to play a role in the early phases of HIV-1 replication as the proviral DNA nuclear import [103] and its deletion causes the enhancement of MA accumulation in the cytoplasm at the expense of PM. On the contrary, Src overexpression was found to promote the localization of Pr55^Gag^ at the PM [102].

In sum, HIV-1 Pr55^Gag^ is phosphorylated by at least three kinases, PKC, ERK-2 and Src. Interestingly, mutation of phosphorylated residues in the p6 domain revealed that this domain, in addition to MA, can act as membrane targeting domain of Gag [104]. However, phosphorylation positions in p6 mainly display redundancy, thus hindering the evaluation of the impact of each individual phosphorylated residue.

## 6. Gag Phosphorylation in Other Retroviruses

Phosphorylation is a conserved modification in the retroviral family (Table 2). In alpharetroviruses, within the RSV MA domain, a small proportion of Y residues results in being phosphorylated [105], as well as S68 and S106 residues (Table 2). However, S68 seems to be transitionally phosphorylated, while S106 is the main phosphorylated signal [106]. Besides, MA phosphorylation could be involved in the recruitment of factors promoting NC phosphorylation [106,107]. In turn, phosphorylation of S529 in NC was found to be necessary for the specific interaction with gRNA [108], but no other notable effects on assembly, or on infectivity, were observed [106].

The deltaretroviruses, HTLV-1 MA is also a phosphoprotein, and S105, which is located close to the two late domains involved in viral release [109], PPPY [110] and PTAP [111], is phosphorylated by ERK-2. Similarly, to HIV-1, ERK-2 is incorporated into HTLV-1 particles, and phosphorylation of the MA domain was found to be involved in virus release and budding efficiency [110].

Interestingly, betaretroviruses such as MPMV encode a phosphoprotein pp24 within the Gag precursor, and its C-terminal cleavage produces the protein pp18 which contains proline-rich motifs (PPPY). Deletion assays indicated that the phosphorylated residue Y205 in pp18 is dispensable for capsid assembly, but is necessary for the viral release [112]. Immunoprecipitation experiments identified the presence of phosphoserines in pp18, [113,114] displaying a redundant character. Similarly, for spumaviruses such as FV, mapping of the p4 domain revealed that seven residues can be phosphorylated (Table 2), but a single substitution of those residues displayed no influence on viral replication [115]. In gammaretrovirus, the phosphorylation of the RNA binding phosphoprotein (p12) within the Gag precursor was found to be necessary for early events of viral life cycle and virion production [116,117]. Mutagenesis experiments identified two residues which can be phosphorylated (S192 and S209). In particular, S192 mainly contributes to p12 phosphorylation and its substitution by A impairs viral assembly and infectivity. However residual phosphorylation can also occur at other positions (Table 2) [117], thus suggesting a redundant character of these modified amino acids. Indeed, the single substitution of one of these residues induced different levels of phosphorylation in p12, displaying no overall effect on the viral cycle [117], even though these PTMs were proposed to modulate p12 early and late functions and p12 viral RNA-binding activity [117,118].

In conclusion, similarly to HIV-1, the kinases PKC and ERK-2 are the main drivers of retroviral Gag phosphorylation. Interestingly, ERK-2 can be incorporated into the viral particle of HTLV-1. Globally, these PTMs generally seem to play a role in viral particle release and in virus infectivity, even though the impact of the phosphorylation rate in retroviral proteins is complicated by the redundancy of phosphorylated positions.

## 7. HIV-1 Pr55^Gag^ Ubiquitination

Another crucial PTM for retroviral infectivity is ubiquitination. This PMT consists of intracellular protein modification by adding one or more ubiquitin (Ub) protein(s) (for a review see [119]). Ub is a 76-amino acid polypeptide, which has a conserved structure [120]. The Ub sequence contains seven K residues that can be used for subsequent Ub linkage leading to polyubiquitination (for a review see [121]), even if the two most common polyubiquitination chains consist in the formation of Ub chain connected to residues K48 or K63 of Ub. Monoubiquitylation corresponds to a signal for DNA repair, and vesicle sorting or signal transduction, while polyubiquitinated proteins are often targeted to the 26S proteasome for degradation, or alternatively involved in regulation of the endocytosis of ESCRT-dependent cargo proteins into Multi Vesicular Bodies (MVB) (for a review see [122]) and DNA damage response [123]. Ubiquitination can be reversed by deubiquitinating enzymes (DUB) [124]. 

**Figure 5:**
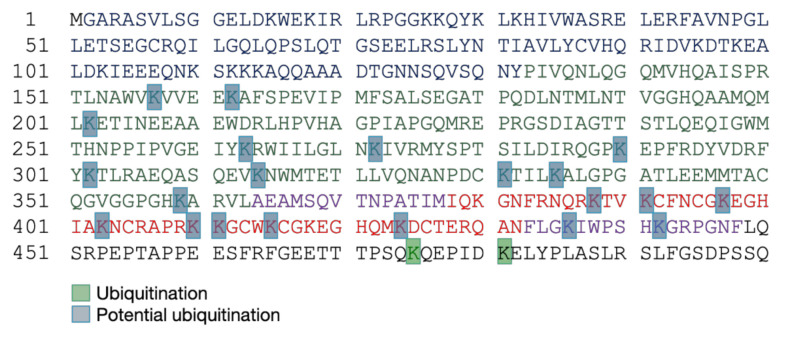
Ubiquitinylated residues in HIV-1 Pr55^Gag^. The domains of Pr55^Gag^ are represented by different colors (see Figure 4). Experimentally identified ubiquitinylated positions are highlighted in light green. Potential ubiquitinylated positions are highlighted in light blue.

HIV-1 Pr55^Gag^ is ubiquitinated in its domains at different levels (Table 3 and Figure 5). Indeed, MA, CA, and NC are monoubiquitinated, while p2 can be mono or bi-ubiquitinated [125]. The cumulative mutations of ubiquitin acceptor sites were observed to cause generally budding defects, even if the substitution of K residues by R in CA (Table 3) revealed very limited effect on viral release, showing that these ubiquitination sites are likely redundant [126]. Besides, it was observed that the level of Pr55^Gag^ ubiquitination increases in cellula when a full-length HIV-1 molecular clone is expressed in comparison to a Pr55^Gag^ expression plasmid, suggesting a role of other viral proteins in Pr55^Gag^ ubiquitination [125]. Globally, the ubiquitination of Pr55^Gag^ was found to be involved in the viral release and, during HIV-1 assembly, viral particles incorporate free Ub proteins corresponding to about 10% of the Pr55^Gag^ level, and around 2–5% of ubiquitinated Pr55^Gag^ are mono-ubiquitinated [125,127,128,129,130]. When the level of free Ub in cells is reduced by proteasomal inhibition, the number of free Ub in viral particles and the number of mono-ubiquitinated residues in the p6 domain of Pr55^Gag^ also decreased [125,127,131]. However free Ub incorporation into viral particles seems to be independent from the global Pr55^Gag^ ubiquitination state [132], and the ubiquitination level in virions increased upon overexpression of free Ub [133]. Furthermore, ubiquitination seems to take place at the PM, and interestingly the level of Pr55^Gag^ mono-ubiquitination was found to be directly correlated with ability of the precursor to bind the PM [134].

The C-terminal p6 domain is the most ubiquitinated domain in Pr55^Gag^ [125], and K475 and K481 are the major targets. Even if these mono-ubiquitinated residues are neither directly involved in virus release, nor in infectivity, they were found to be necessary to promote the overall ubiquitination of Pr55^Gag^ [132]. Besides, the mutation of the highly conserved and phosphorylated S488 residue in p6 domain with F (S488F), which can occur spontaneously during anti-retroviral treatments, has not only an impact on virus morphogenesis, maturation and virion infectivity (Table 1) [87,88,89], but it can also induce conformal changes in p6, resulting in an enhanced interaction of Pr55^Gag^ with the PM. This would lead to the polyubiquitination of the precursor and consequently to its proteasomal degradation [104].

The p6 domain is known to be involved in the recruitment of host factors, such as Tsg101 (Tumor susceptibility gene 101) and ALIX (ALG-2 interacting protein X), and ubiquitination of those factors strongly promote viral budding [135]. In this frame, fusion experiments in which the p6 domain was coupled with Ub showed that the affinity of Tsg101 for p6 in this case results in being strengthened [136], and the ubiquitination of Pr55^Gag^ can increase Tsg101 recruitment [137]. Besides, Tsg101 displays an N-terminal Ub E2 variant (UEV) domain that shows homology with E2 Ub ligases, and that can specifically bind Ub proteins, as well the PTAP late domain in Pr55^Gag^ [136]. During assembly, the interaction of Pr55^Gag^ with the PM promotes the intermolecular interaction between Tsg101 and the PTAP domain in Pr55^Gag^ [137]. In this conformation, the di-ubiquitinylated K63 of Tsg101 was found to interact with p6, with the consequence of impairing the potential polyubiquitination of the precursor at PM [137].

Finally, the ESCRT-III-associated ALIX protein is also ubiquitinated [128] and specifically interacts with the E3 ubiquitin-protein ligase NEDD4 that can bind the proline-rich retroviral domain PPPY. The interaction between NEDD4 and the retroviral precursor leads to the recruitment of the ESCRT-III complex, including the eukaryotic sucrose non-fermenting protein 7 (Snf7), and the vacuolar protein sorting-associated proteins Vps 2, Vps20 and Vps24, and Vps4 in order to promote retroviral release [138,139,140,141]. Since in HIV-1 the PPPY domain is absent, ALIX recruits directly NEDD4 to facilitate this step [128,129].

## 8. Gag Ubiquitination in Other Retroviruses

The role of ubiquitination in the retroviral cycle is not yet fully elucidated. Some retroviruses display a functional contribution of Ub modifications in virus release such as MLV, MPMV or RSV, and for those viruses, it was shown that, similarly to what observed for HIV-1 [131], the inhibition of proteasome not only induces a reduction of the level of free Ubs in the cytoplasm, but also impairs the release of the viral particles (Table 4) [127,132,133,142]. In addition, fusion experiments between RSV Pr76^Gag^ and Ub, or overexpression of Ub, displayed an increase in viral particle release [142], thus supporting the idea that ubiquitination of retroviral precursors is crucial for viral budding [133]. However, for other retroviruses such as MMTV or HTLV-1, to date it was not possible to identify a precise role of ubiquitination [127] (Table 4). Besides, the inhibition of the proteasome did not impair the budding of EIAV [127,143]. On the other hand, similarly to HIV-1, EIAV particles contain free Ubs corresponding to 10–15% of Gag proteins. Likewise, the C-terminal p9 domain is mono-ubiquitinated and contains a YPDL late domain which is involved in the recruitment of the ESCRT machinery [143,144]. Moreover, p9 also contains an Ub-like motif (NVKEKD) that may contribute to virus release, thus suggesting alternative release pathways for EIAV even if Ub quantity is low [133,144].

MMTV Gag is monoubiquitinated in its p8 domain, in CA, and is potentially di-ubiquitinated in NC [127]. In comparison with other retroviruses, MMTV does not contain late domains such as PPPY and PTAP, but an alternative PSAP late domain was found in CA, although its functional role was not yet elucidated. Besides, YXXL motifs, which also represent alternative late domains, were identified in MA and in pp21 viral factor. Importantly, since Gag ubiquitination seem to take place mostly in regions close to the late domains [133], it is possible that the presence of these alternative late domains in EIAV and MMTV precursors promote virus release.

In HLTV-1, more than 40% of MA are ubiquitinated [111,145,146], and MA can be mono- and di-ubiquitinated [146]. Furthermore, mutagenesis experiments identified K74 in Pr53^Gag^ as the main substrate for ubiquitination [146]. Indeed, substitution experiments in which the K74 is replaced by an R resulted in a decreased release of infectious particles [146]. The ubiquitination of K74 could also play a role in the recruitment of NEDD4 [146], which is also involved, through its interaction with the PPPY late domains, in the release of other retroviruses such as MMPV [112], avian sarcoma virus (ASV) [147], and MLV [132,148].

In RSV particles, more than 100 free Ubs were found [142,149]. However, contrary to other retroviruses, RSV displays free Ubs exclusively into mature viruses [149]. Since Pr76^Gag^ mono-ubiquitination was found to be necessary for budding and to recruit the ESCRT machinery [142,149], it is thus possible that the presence of free Ubs could be the result of a host-encoded and encapsidated deubiquitinating enzyme (DUB) [124]. Interestingly, this process was also observed to occur during budding or cells lysis [124]. Finally, Gag precursors from spumavirus encode a very limited number of K residue. This observation suggests that Gag of spumavirus could not be a favorable substrate for the ubiquitination machinery [150].

## 9. HIV-1 Pr55^Gag^ Sumoylation

**Figure 6:**
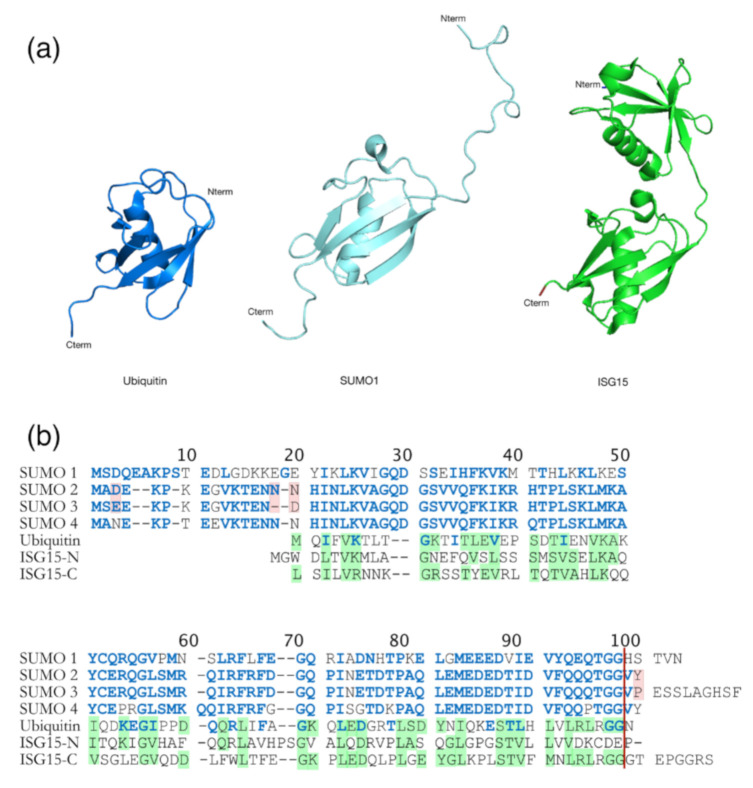
Comparison between Ub and Ub-like proteins: SUMO and ISG15. (**a**) Structural comparison between Ub (heavy blue, PDB: 1A5R), SUMO-1 (ligth blue, PDB: 2QHO), and ISG15 (green, PDB: 3PHX). They contain a typical ββαββαβ fold, even if SUMO-1 has long unstructured N-terminal domain which is absent in Ub. ISG15 is composed with two Ub-like domains in N- (TSG15N) and C- (TSG15C) terminus. (**b**) Amino acid sequence alignments of Ub, the four SUMO homologs and ISG15 from humans. Identities and similarities are indicated between Ub and SUMO (blue residues into Ub sequence) and between Ub and ISG15 (shaded green residues in Ub and ISG15). Differences between SUMO-2 and 3 are highlighted in pink. The red vertical line represents the GG end free after the maturation step required for sumoylation. The amino acid sequence homology between SUMO and Ub is 18% [152], and 30% between Ub and ISG15 [161].

Another modification important for retroviral infectivity is sumoylation, which is a reversible PTM and consists of intracellular protein modification by a covalently attached small Ub related modifier (SUMO) protein to a K substrate (for reviews see [151,152]). Even though SUMO is structurally comparable to Ub (Figure 6a), it presents many differences in amino acids sequence (only 18% of homology) (Figure 6b) [152]. This PTM is usually involved in the maintenance of genomic integrity, with a role in repair of damaged DNA, and in the regulation of transcription and in gene expression. Like ubiquitination, sumoylation is involved in intracellular signal transduction and can regulate biological processes such as apoptosis, immune response, and carcinogenesis. Besides, sumoylation controls protein localization and it can induce protein conformational changes. SUMOs are highly conserved in eukaryotes, and four SUMO isoforms (SUMO-1 to SUMO-4) are present in mammals [152,153,154] (Figure 6b). Similarly, to Ub, the C-terminus region of SUMO-1 is linked to ε-amino groups of K residues in the target protein [155,156]. SUMO-1 was interestingly found to counterbalance the effect of ubiquitination [157]. SUMO-2 and SUMO-3 are mainly involved in the cellular response to environmental stresses [156] and display very similar sequences with more than 95% identity [151,152,156]. For this reason, they are often named SUMO-2/3. Finally, SUMO-4 is less well known, and its mRNA had been found in few organs such as kidney, spleen, and lymph nodes [152].

**Figure 7:**
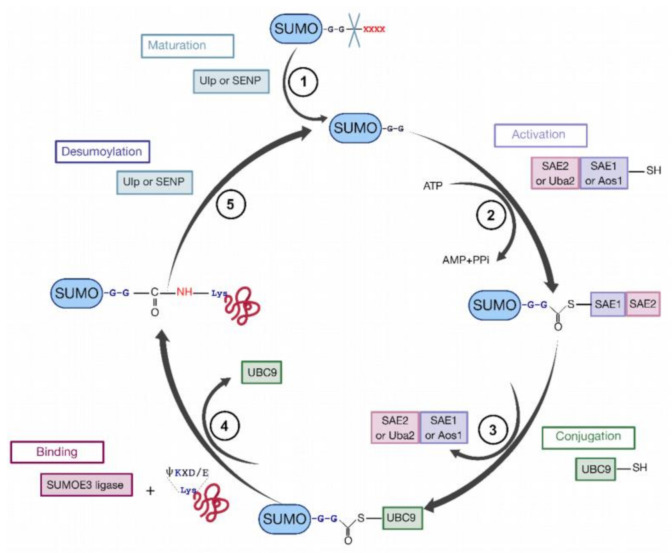
The cycle of sumoylation. This modification is catalyzed by different enzymes and consists in ligation of SUMO protein to K residues of protein substrates. (**1**) SUMO is maturated by Ub-like specific protease 1 (Ulp1) or human sentrin-specific protease 1 (SENP1). This proteolytic cleavage exposes the C-terminal GG motif required for the activation step. (**2**) SUMO is activated by a heterodimer composed with SAE1/SAE2 (Aos1/Uba2) to form the SUMO (E1/E2)-activating enzyme. Heterodimer is bound via a thioester bond between the C-terminal G residue of SUMO and the catalytic C of SAE2. (**3**) SUMO is transferred to the catalytic C of SUMO-conjugating enzyme E2 (or Ubc9) by a transesterification reaction. (**4**) SUMO is bound to the target protein by Ubc9 in association with SUMO E3 ligase. Ubc9 forms an amide bond between the SUMO C-terminus and ε-amino groups of the acceptor L residues in the target protein. (**5**) These reactions are reversible by means of the Ulp or SENP proteases.

To sumoylate a protein, different successive biochemical reactions are required [152,158,159,160] (Figure 7). Generally, the consensus sequence for K sumoylation is ψKXD/E (ψ stands for a hydrophobic residue). Nevertheless, targets with non-consensus acceptor sites have also been identified [151,152].

The p6 domain of HIV-1 Pr55^Gag^ is sumoylated by SUMO-1, which covalently links K475 in the consensus sequence (ψKXE: QKQE) (Table 5). The K475R substitution partially inhibits binding of the precursor to the SUMO-conjugating enzyme E2 (Ubc9) [162], suggesting that more than one Pr55^Gag^ domain could be involved in the recruitment of Ubc9 [162,163]. It was proposed that SUMO-Ubc9 could be involved in intracellular trafficking of Pr55^Gag^ [164]. Indeed, after translation, the first trafficking complex intermediate observed in the perinuclear region is composed of Pr55^Gag^, kinesin family motor 4 (KIF4), Ubc9, and SUMO-1 [164]. In contrast, other studies suggested that the recruitment of Ubc9 would be required for the late stages of viral replication, thus participating to Env incorporation into viral particles [163]. Moreover, the overexpression of SUMO-1 was observed to globally decrease viral infectivity, and sumoylation could be then involved in the negative regulation of viral replication [162]. Interestingly, sumoylation and ubiquitination co-regulate each other [165], and sumoylation and mono-ubiquitination of p6 were both found to occur on K475. It is thus possible that SUMO-1 interaction with p6 protects Pr55^Gag^ from proteasomal degradation [162]. Overproduction of SUMO-1 should have no direct effect on viral assembly, but if sumoylation competes with ubiquitination, subsequent decrease of Tsg101 recruitment could produce a negative effect on budding [162].

## 10. Gag Sumoylation in Other Retroviruses

As for HIV-1, other retroviruses are sumoylated (Table 5); however, the impact of sumoylation is not yet fully elucidated. The Ubc9 factor was found to interact with MLV and MPMV Gag proteins [166,167]. Similarly, to HIV-1, in MPMV this factor was suggested to be involved in the trafficking of Pr78^Gag^ to the PM [167]. Besides, the CA domain of MoMuLV Gag was shown to interact not only with Ubc9 [168], but also with PIASy, a SUMO E3 ligase [168]. These interactions, leading to CA sumoylation during the early stages of the viral life cycle after reverse transcription, might have a role in viral replication [168]. Single K substitutions have generally no effect on the viral cycle, suggesting redundancy between sumoylable positions. On the other hand, the modification of K218 with an R residue was found to reduce the overall viral replication, without affecting the overall SUMO-1 rate on Gag [168].

In the EIAV p9 domain of Gag, K465 is the main target for sumoylation, and mutational experiments showed that this PTM is involved in the regulation of viral replication and infectivity [144,169]. Moreover, sumoylation of K465 seems to regulate the sumoylation of other K residues in different domains of the precursor, such as the MA, the CA and NC (Table 5) [169]. However, a specific role of all those PTMs in viral replication remains to be clarified. Finally, K244 in CA of RSV Pr76^Gag^ was found to be sumoylated, and its substitution with a non-sumoylable R residue (K244R) displayed decreased viral infectivity [142].

Similarly, to ubiquitination, the exact role of sumoylation is still a matter of debate and might be different among retroviruses. Moreover, sumoylation is still very difficult to detect, and thus further technological advances will be required to better identify and characterize this PTM.

## 11. Retroviral Gag Protein ISGylation

**Figure 8:**
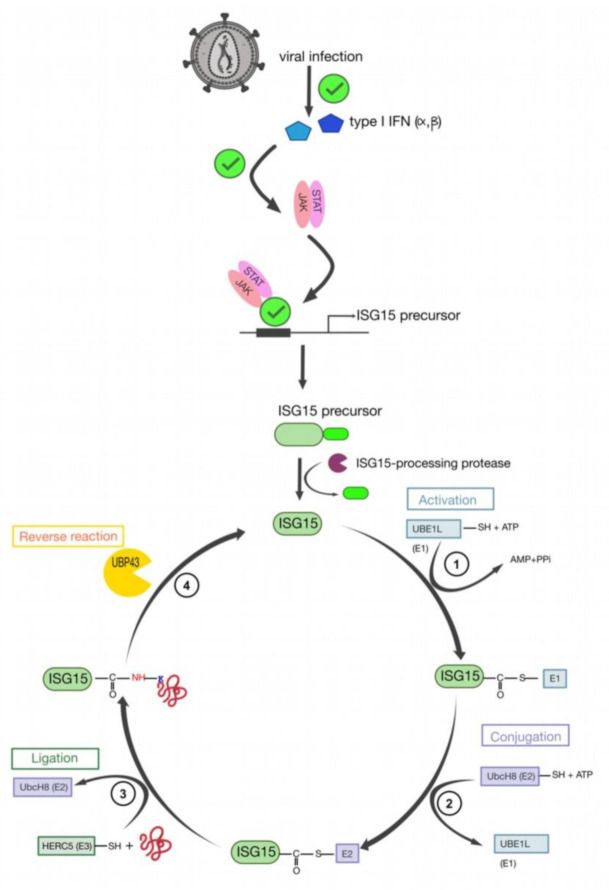
The cycle of ISG15. Viral infection induces the expression of type I IFN. These molecules activate the JAK/STAT signaling pathway, which is responsible for the activation of the ISG15 promoter (for reviews see [161,172]). The ISG15 is maturated by ISG15-specific proteases which cleave the C-terminal extension from ISG15 precursor. (**1**) The mature ISG15 is activated by UBE1L (E1). It corresponds to the formation of a thioester bond between ISG15 and E1. (**2**) ISG15 linked to UBE1L is transferred to UbcH8 (E2). (**3**) Finally, E2 recruits an E3 ligase such as HERC5, transferring the activated Ub from the E2 to the K substrate (ligation reaction). (**4**) The reaction can be reverted via UBP43. Indeed, it cleaves ISG15 molecules that are conjugated to the substrate proteins via isopeptide bonds (adapted from [161]).

Besides sumoylation, there exists another Ub-like protein, which is the interferon stimulated gene 15 (ISG15) (for reviews see [161,170]). ISG15 was identified in mammals and corresponds to a 17 kDa protein induced by type I IFN (α and β) ([171,172], for a review see [173]) (Figure 8) that contributes to regulation of the cell cycle, and plays a role in stress response, signaling transduction, and immune response. The IFN response starts with the binding of type I IFN to cell specific receptors, leading to the activation of the Janus kinase (JAK), the signal transducers and the activators of transcription (STAT) signaling pathway (for a review see [174]), which stimulate the transcription of several hundreds of ISGs, including ISG15. This last one presents a sequence homology with Ub, as it contains two Ub-like domains (Figure 6b). The cycle of ISGylation is comparable with the one of Ub (Figure 8). Indeed, three distinct biochemical reactions leads the binding between ISG15 and the target protein. This reaction is reversible and the Ub specific peptidase 18 (USP18), also called UBP43, is involved in the reverse reaction, thus acting as an ISG15 deconjugating enzyme [175].

Interestingly, recent studies on HIV-1 gave first information about the role of ISG15 in viral replication. Indeed, suppression of IFN produced by dendritic cell (pDC2) induces the rapid progression of viral infection [176], thus displaying an antiviral role for ISG15. Moreover, in vitro studies showed that ISG15 would inhibit not only the early [177,178] but also the late steps [179,180] of viral replication. The co-transfection with plasmids expressing ISG15 and HIV-1, inhibited the release of viruses, while no impact was seen on HIV-1 proteins production [181]. Moreover, the overexpression of ISG15 and of the activating enzyme UBE1 was observed to impair HIV-1 replication [181]. Interestingly, in these assays, the ubiquitination of Pr55^Gag^ and Tsg101 also resulted affected, with the consequent abolition of the interactions between the p6 late domain and Tsg101. This impairs the assembly of viral particles and their release, and EM assays showed that immature viruses accumulate of at the PM [180,181]. Furthermore, the production of viral particles was also found to be impaired by the E3 ligase HERC5. Interestingly, this inhibition was not found to alter the trafficking of HIV-1 Gag to the PM, but the budding at the PM [180]. Since HERC5 is described to restrict also MLV Gag particle production, it results that HERC5 and more generally, the response ISG15, can be considered as a restrictive factor against retrovirus [180].

**Figure 9:**
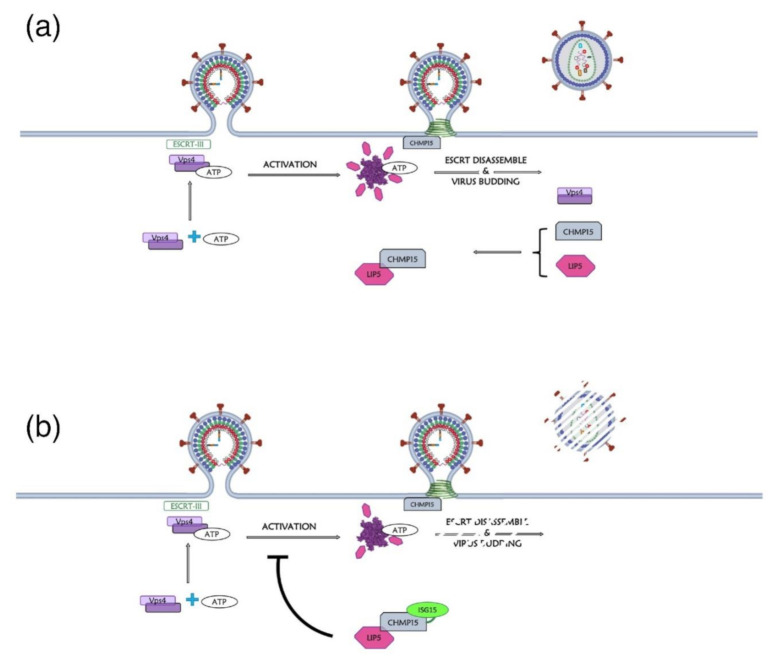
Model of the impact of ISG15 on Vps4 function during retroviral budding. (**a**) Normal assembly and budding phase during the retroviral cycle. Vps4 activity depends on its oligomeric state. In its dimeric form, Vps4 is cytosolic and inactive. During retrovirus assembly at PM, upon polymerization of the ESCRT-III complex with the p6 domain of HIV-1 Pr55^Gag^, ATP-bound Vps4 is recruited at the PM. At this step, Vps4 interacts with the coactivator protein LIP5, which is bound to CHMP5, and achieves its double hexameric-ring structure. Then, ATP hydrolysis by the Vps4-LIP5 oligomer releases the ESCRT-III complexes from PM and the dissociation of the ESCRT complex coincides with the membrane fission event that releases retrovirus particles. (**b**) ISG15 inhibits the budding phase. When CHMP5 is ISGylated, this prevents the interaction between Vps4 and LIP5 by excluding LIP5. In the absence of the Vps4-LIP5 complex, the ESCRT-III complex remains trapped at the PM and the viral release is thus impaired (adapted from [179]).

In general, ISG15 may affect many other RNA viruses and other retroviruses such as the avian sarcoma leukosis virus (ASLV) (for review see [182]). Similar to HIV-1, ISG15 inhibits the release of ASLV, and the ubiquitination of ASLV Gag precursor. In this context the E3 ubiquitin-protein ligase, NEDD4 was found to maintain its ability to bind the late motif in ASLV Gag [179]. Thus, ISG15 does not seem to prevent directly the interaction between NEDD4 and ASLV Gag, but likely it interferes with the Ub ligase activity of NEDD4, which inhibits ubiquitination [183] even if the precise mechanism remains unclear. Alternatively, it was proposed that the impaired budding of ASLV and HIV-1 could be due to the ISGylation of the ESCRT-III component Charged Multivesicular body Protein 5 (CHMP5) (Figure 9a,b). Indeed, during retroviral budding, the ESCRT-III complex polymerizes at PM in interaction with Pr55^Gag^. This complex then recruits the inactive dimer form of the ATPase Vps4, which requires it to recruit ATP and its coactivator protein, Vps-associated protein LIP5, to achieve its activated double hexameric-ring structure. This leads to the disassembly of the ESCRT-III complex, thus promoting the viral budding (Figure 9a). According to the proposed model, ISGylation of CHMP5 was found to impair Vps4 binding to LIP5, and thus Vps4 would remain in its inactivated conformation while ESCRT-III complexes would be trapped at the PM, thus blocking virus budding [179] (Figure 9b).

Similar mechanisms can occur in the context of other retroviruses [179].

## 12. Post-Translational Methylation of Retroviral Gag Proteins

Finally, retroviral Gag precursors are also subjected to methylation. This covalent PTM is reversible [184]. It consists of the transfer of a methyl group from a donor, the S-adenosylmethionine (SAM), to a target K residue, and this reaction is catalyzed by the K-methyltransferases (for reviews see [185,186]). The same residue can be mono-, di- or tri-methylated, thus conferring a signature which can be specifically recognized by transacting factors named “readers”, whose recruitment can promote signaling pathways, regulation of protein–protein interactions, transcription, T-cells activation [187] and subcellular localization [188]. Interestingly, immunoblotting of the CA domain of HIV-1 in presence of AdOx, an inhibitor of methylation, showed an increase of mature CA, suggesting that methylation of HIV-1 Pr55^Gag^ could affect proteolytic maturation and likely the viral assembly [189]. The basic region of the NC domain of HIV-1 Pr55^Gag^ is also methylated, and this modification was proposed to be involved in the subnuclear localization of the precursor [190]. This same PMT on NC would also decrease the rate of tRNA^Lys3^ annealing to the PBS region in gRNA, thus inducing defects in the reverse transcription step [191]. Similarly, the R540 residue in the NC domain of PFV Pr74^Gag^ was found to be methylated, and this modification seems to be required for the subnuclear localization of the precursor [192].

## 13. Conclusions

**Figure 10:**
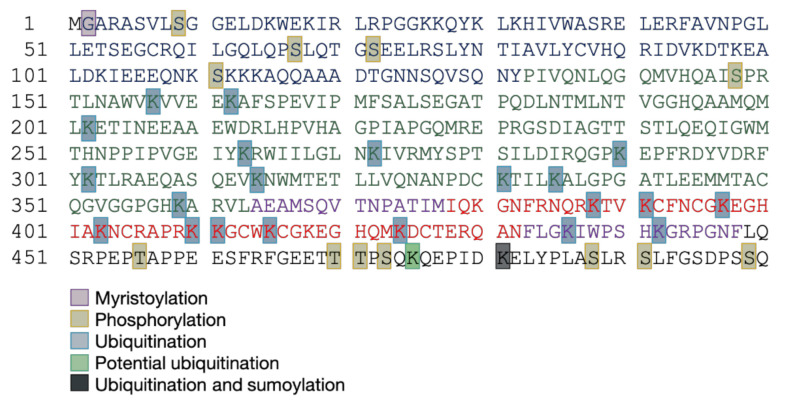
Summary of post-translational modifications of HIV-1 Pr55^Gag^ residues. The domains of Pr55^Gag^ are represented by different colors (see Figure 4). Experimentally identified modified residues are highlighted: myristoylation (pink), phosphorylation (yellow), ubiquitination (light blue), potential ubiquitinations (light green), and sumoylation (black).

PTMs create a vast diversity in proteins and thus regulate their functions. Globally, PTMs play a role in many processes such as cell signaling, and protein–protein and protein–RNA interactions. Besides, PTMs are crucial for the life cycle of many viruses and the characterization of viral PTMs would provide a better understanding of the mechanisms of viral processes. HIV-1 Pr55^Gag^, as many other retroviral Gag precursors, displays several PTMs in its different domains (Figure 10). These PTMs include myristoylation, phosphorylation, ubiquitination, sumoylation, and methylation. All these PTMs can have either antagonistic or cooperative roles, thus allowing fine regulation of the viral cycle. However, up to now, the role of many of these modifications is not fully elucidated and further investigations will be required to better understand their contributions in the viral life cycle. One of the main challenges to study PTMs carried by proteins consists of the development of refined proteomic technologies, allowing the specific detection and characterization of the modifications. The improved knowledge of those regulations would be useful in the future to identify new targets for antiretroviral treatments.

## Figures and Tables

**Table 1:** Summary of different roles of phosphorylated residues in HIV-1 Pr55^Gag^.

Domain	Residue	Enzyme	Observations and Associated (or Proposed) Roles	References
**MA**	S9	ERK2	Involved in the viral replication Phosphorylation of the mature form of MA	[95,101]
	S67			
	S72			
	S77			
	S111		PKC could be involved in membrane binding by regulating the exposure of the myristoyl group	[82,83,84]
	Y132	Src	In MA mature 1% of Y132 is phosphorylated Src overexpression was found to promote the localization of Pr55^Gag^ at the PM	[102,103]
**CA**	S148	ERK2	Belongs to S-P motif involved in recruitment of ERK-2	[93,94,95,96]
**p6**	T456		Belongs to the PTAP late domain Potential role in viral infectivity and assembly	[86]
	T470		Redundancy with T471, S473, S488, S491, and S499	[91]
	T471	ERK-2	Belongs to T-P motif involved in the recruitment of ERK-2	[91]
			Its substitution induces the accumulation of immature viral particles incompletely separated from PM	[91]
			Redundancy with T470, S473, S488, S491, and S499	[91]
			Effects on assembly or on viral release is not due to phosphorylation	[86]
	S473		Redundancy with T470, S471, S491, and S499	[91]
	S488	ERK2	Viral particles without active ERK2 were found to be poorly infectious due to a defect in reverse transcription	[93,95]
			Involved in the phosphorylation of other viral proteins: Rev, Nef, Vif, mature MA	[97,98,99,100]
		PKC	The p6 domain of Pr55^Gag^ is a target for PKC	[85,86,90]
			The inhibition of PKC activity reduced Vpr level in virions	[85,87,88]
			Its mutation with F perturbs: - Viral morphology, maturation and infectivity	[87,88,89]
			Effects on assembly or on viral release could be not due to phosphorylation	[86]
	S491		Redundancy with T470, S471, S473, and S499	[91]
	S499		Redundancy with T470, S471, S473, and S491	[91]

**Table 2:** Summary of phosphorylated positions in the different domains of retroviral Gag precursors.

Retrovirus	Protein	Residues	Enzyme	Observation and Associated (or Proposed) Roles	References
**RSV**	MA	Y15	PKC	No effect on the viral cycle	[105,106]
		Y46			
		S68			
		S106	PKC	Major site of phosphorylation Involved in the recruitment of factors which promote NC phosphorylation	[105,106]
		Y155	PKC	No effect on the viral cycle	[105,106]
	NC	S529		Role for the specific interaction with the gRNA	[106,107]
**HTLV-1**	MA	S105	ERK2	Close to late domains (PPPY et PTAP) Involved in viral release and budding efficiency	[110]
**MoMuLV**	p12	S137		- Redundancy - Modulation of early and late functions and the RNA-binding activity of p12	[117]
		S148			
		S150			
		S173			
		S192		- S192 mainly contributes to p12 phosphorylation and its substitution by A impairs viral assembly and infectivity	[117]
		S209			
	
**MPMV**	p18	Y205		Belongs to proline-rich motif (PPPY) Necessary for the viral release	[112]
		S167		Redundancy	[113,114]
		S176			
		S211			
**FV**	p4	S116		Redundancy	[115]
		S119			
		S120			
		S124			

**Table 3:** Summary of ubiquitinations in HIV-1 Pr55^Gag^ proteins.

Domain	Residues	Observation and Associated (or Proposed) Roles	References
**MA**		Mono-ubiquitination	[125,126]
**CA**	K157 K162 K202 K263 K272 K290 K302 K314 K331 K335 K359	Mono-ubiquitination Observed redundancy	[125,126]
**NC**	K388 K391 K397 K403 K410 K411 K415 K424	Mono-ubiquitination	[125,126]
**p2**	K436 K442	Mono or di-ubiquitination	[125,126]
**p6**		Mono or di-ubiquitination. Most ubiquitinated domain in Pr55^Gag^	[125,126]
	K475	Major target for mono-ubiquitination No effect in the viral release and infectivity Involved in global Pr55^Gag^ ubiquitination	[125,132]
	K481	Major targets for mono-ubiquitination No effect on virus release and infectivity Involved in the global Pr55^Gag^ ubiquitination	[125,132]
	S488F	Conformal changes: formation of a hydrophobic patch in a-helix at the C-terminus of p6 Leads to strong interaction of Pr55^Gag^ with the PM This structure promotes L48 linked polyubiquitination	[104]

**Table 4:** Summary of ubiquitinations in the different domains of retroviral Gag proteins.

Retrovirus	Domain	Residues	Associated (or Proposed) Roles	References
**HIV**			About 100 free Ubs are incorporated into viral particles 2–5% mono-ubiquitinated	[130,132,133,134]
			Pr55^Gag^ ubiquitination promotes the virus release K475 and K481 in p6 domain are major targets for ubiquitinations Pr55^Gag^ ubiquitination is correlated with the ability of the precursor to bind the PM	[132,134]
**MLV**			Increases viral release and infectivity	[127]
	p12		PPPY late domain is involved in the recruitment of NEDD4	[148]
**HTLV-1**	MA		Ubiquitination of this domain has a crucial role in release	[111,145,146]
			40% of MA are ubiquitinated MA can be mono- or di-ubiquitinated	[111,145,146]
		K74	Substrate for Pr53^Gag^ ubiquitination	[146]
**MPMV**			PPPY late domain is involved in the recruitment of NEDD4	[112]
**RSV**			- Mono-ubiquitination is crucial for viral release - Ubiquitylation is required for the recruitment of ESCRT machinery and for the budding	[133,143,149]
			- Contains free Ubs into mature particles - Pr76^Gag^ mono-ubiquitination is necessary for budding and to recruit the ESCRT machinery	[142,149]
**EIAV**			10–15% of the molar level of the Gag protein of free Ub	[143]
			Proteasome inhibition: does not impair the release	[143]
	p9		Ub-like motif (NVKEKD)	
			Mono-ubiquitinated domain Contains YPDL late domain	[143,144]
**MMTV**			Proteasome inhibition: does not decrease the release	[143]
	MA (p10)		YXXL Late domain	[127]
	pp21		YXXL Late domain	[127]
	p8		Mono-ubiquitinated	[127]
	CA (p27)		Mono-ubiquitinated	[127]
			PSAP domain	[127]
	NC (p14)		Di-ubiquitinated	[127]
**PFV**			Encodes for a very restricted number of K residues	[150]

**Table 5:** Summary of sumoylated positions in retroviral Gag proteins.

Retrovirus	Domains	Residues	Associated (or Proposed) Roles	References
**HIV-1**			Sumoylation and ubiquitination co-regulate each other	[165]
	p6		More than one domain should be involved in Ubc9 recruitment	[162,163]
			SUMO-Ubc9 could be involved in intracellular trafficking of Pr55^Gag^ - in the early phase: perinuclear region or - in the late stages of replication: potential role in Env incorporation	[163,164]
		K 475	Sumoylation could be then involved in the negative regulation of viral replication	[162]
			Belongs to QKQE consensus sequence Sumoylation and mono-ubiquitination of p6 can both occur on K475	[162]
**MoMuLV**	CA		CA domain of MLV Gag interacts with Ubc9 and with PIASy	[168]
**MPMV**			Recruitment of Ubc9 involved in the active transport of MPMV Pr78^Gag^ to the PM	[167]
**RSV**	CA	K 244	Its substitution with non sumoylable R reduces the overall viral infectivity	[142]
**EIAV**	MA	K 13 K 86 K 107	Targets of sumoylation	[144,169]
	CA	K 282 K 297		
	NC	K 368 K 373 K 388 K 420 K 423		
	p9	K 465	Constitutes the main target for sumoylation	[144,169]

## Data Availability

All figures and discussed literature are available in the main text of this review.
